# Using simple agent-based modeling to inform and enhance neighborhood walkability

**DOI:** 10.1186/1476-072X-12-58

**Published:** 2013-12-11

**Authors:** Hannah Badland, Marcus White, Gus MacAulay, Serryn Eagleson, Suzanne Mavoa, Christopher Pettit, Billie Giles-Corti

**Affiliations:** 1 McCaughey VicHealth Centre for Community Wellbeing, School of Population and Global Health, University of Melbourne, Melbourne, Australia; 2 Melbourne School of Design, Faculty of Architecture, Building, and Planning, University of Melbourne, Melbourne, Australia; 3 Computing and Information Systems, University of Melbourne, Melbourne, Australia; 4 Centre for Spatial Data Information and Land Administration, Faculty of Engineering, University of Melbourne, Melbourne, Australia; 5 McCaughey VicHealth Centre for Community Wellbeing, School of Population and Global Health, University of Melbourne, Melbourne, Australia; 6 Australian Urban Research Infrastructure Network & Faculty of Architecture, Building, and Planning, University of Melbourne, Melbourne, Australia; 7 McCaughey VicHealth Centre for Community Wellbeing, School of Population and Global Health, University of Melbourne, Melbourne, Australia

**Keywords:** AURIN, Catchment modeling, Health, Liveability, Public transport, Schools, Spatial data, What-if

## Abstract

**Background:**

Pedestrian-friendly neighborhoods with proximal destinations and services encourage walking and decrease car dependence, thereby contributing to more active and healthier communities. Proximity to key destinations and services is an important aspect of the urban design decision making process, particularly in areas adopting a transit-oriented development (**TOD**) approach to urban planning, whereby densification occurs within walking distance of transit nodes. Modeling destination access within neighborhoods has been limited to circular catchment buffers or more sophisticated network-buffers generated using geoprocessing routines within geographical information systems (**GIS**). Both circular and network-buffer catchment methods are problematic. Circular catchment models do not account for street networks, thus do not allow exploratory ‘what-if’ scenario modeling; and network-buffering functionality typically exists within proprietary GIS software, which can be costly and requires a high level of expertise to operate.

**Methods:**

This study sought to overcome these limitations by developing an open-source simple agent-based walkable catchment tool that can be used by researchers, urban designers, planners, and policy makers to test scenarios for improving neighborhood walkable catchments. A simplified version of an agent-based model was ported to a vector-based open source GIS web tool using data derived from the Australian Urban Research Infrastructure Network (**AURIN**). The tool was developed and tested with end-user stakeholder working group input.

**Results:**

The resulting model has proven to be effective and flexible, allowing stakeholders to assess and optimize the walkability of neighborhood catchments around actual or potential nodes of interest (e.g., schools, public transport stops). Users can derive a range of metrics to compare different scenarios modeled. These include: catchment area versus circular buffer ratios; mean number of streets crossed; and modeling of different walking speeds and wait time at intersections.

**Conclusions:**

The tool has the capacity to influence planning and public health advocacy and practice, and by using open-access source software, it is available for use locally and internationally. There is also scope to extend this version of the tool from a simple to a complex model, which includes agents (i.e., simulated pedestrians) ‘learning’ and incorporating other environmental attributes that enhance walkability (e.g., residential density, mixed land use, traffic volume).

## Background

In the last decade, a growing body of evidence demonstrates pedestrian-friendly neighborhoods encourage walking for both recreation and transport [1,2]. This is important, since physical inactivity is the fourth leading contributor to the burden of disease globally [3] and increasing physical activity is an international priority [4]. Specific built environment attributes, such as residential density, street connectivity, and land use mix, either considered separately or together in a commonly combined ‘walkability index’ [5], have been associated with walking for transport and recreation in the local environment [6-8]. Derived using geographical information systems (**GIS**) applied to existing spatial datasets, applications of these walkability attributes include: identifying neighborhoods of ‘high’ and ‘low’ walkability [9]; providing information on the walkability characteristics of a given region [10]; and generating a standardized benchmark to compare different settings in terms of characteristics shown to promote walking [5]. Accordingly, the development and application of the walkability index has yielded many insights into the relationship between urban form and walking behaviors, and helped to guide the public health research agenda [6,7,11,12]. However, the relative inflexibility of the walkability index (i.e., derived using static data sources) means that it has been primarily limited to assessing the ‘walkability’ of existing environments and the street connectivity component is more suited to regional-scaled analysis.

Another factor that impacts on walking, particular walking for transport, is proximity to services or features of interest [13]. This is gaining attention in the literature [14,15], with smart growth and new urbanism principles focusing on transit-oriented developments (TOD). TOD strategies center on creating walkable, denser built environments within a 10-minute walk (or half mile radius) around major transit stations [16]. However, methods for assessing proximity to destinations, such as distance to railway stations and schools, have been limited [17] with one of the simplest and most frequently used methods being a Euclidian ‘circular catchment’ or ‘circular buffer approach’ [18]. A Euclidian buffer is rapid to apply, but does not take into account street network connectivity or barriers to walkability such as major roads, rivers or railway lines with few crossing points, and tends to overestimate catchment areas [19]. As such, it has drawn criticism in the field of planning [17], and is very limited when it comes to assessing ‘what-if’ modeling scenarios. A more accurate approach to catchment analysis is the service area approach, sometimes called ‘isochrone mapping’ or a ‘pedshed’, which can be calculated in contemporary GIS software such as ESRI’s ArcGIS™ with the Network Analyst™ extension. Typically generated as a ratio, this is a useful measure for summarizing the walkability of a given area [20]. This method has greater accuracy than the circular catchment method as it considers street network permeability, natural and man-made barriers, and allows for ‘what-if’ scenario testing [18]. A downside of this method, however, is the software required can be expensive, as it usually requires high-end GIS software. This technique also requires specialist staff, as it is generally too complex for non-expert users. Open-source solutions such as Quantum GIS plus additional plugin software alleviate cost limitations, but can be more challenging from an operational perspective [21].

Although useful, both the walkability index and the circular catchment tools have restricted utility for modeling the impact different built environment scenarios, including TOD, might have on localized walkability, prior to developing or retrofitting areas of interest. For example, modifying the street network, changing traffic light phasing, or access points to features of interest will likely affect the permeability and accessibility of neighborhoods; and there are substantial advantages to simulating and testing different built environment layouts prior to constructing any interventions.

Agent-based modeling is an approach to examining complex systems of autonomous individual ‘agents’. Agents are programmed with rule-based behaviors to interact with each other and their environment over time [22], thereby enabling the system to be analyzed as a whole. Depending on purpose, agents can be programmed with simple (i.e., follow rules and behaviors, interact with the environment) or complex (i.e., learn rules and behaviors, interact with each other) behaviors [23]. As agents are released from a central node through the network, their simulated behaviors can be visualized and observed, including the distances traveled and any junctions that cause congestion. Although agent-based modeling is not new [24], it has become increasingly popular in assessing human movement in the past decade [25,26] due to both software and hardware development [22]. High-end proprietary agent-based modeling software (e.g., Legion™, SWARM™, MASON™) have been used to model complex human behaviors in crowded railway stations and large sports stadiums to predict effectiveness of egress in the event of an emergency, such as a building fire [27,28]. This kind of agent-based modeling is highly detailed and involves programming complex agent behaviors; it is well suited to building-scaled analysis, but is perhaps overly detailed for precinct-scaled analysis. For this reason, our work sought to develop a simplified open-source simple agent-based modeling tool that combined the benefits of service area approach mapping with other dynamic factors related to walkability within precinct catchment areas. These user-specified manipulations included: having the simple agents (i.e., simulated pedestrians) navigate the street network for specified distances or times; applying differing wait times at crossing points; and allowing for diverse agent functionality (e.g., walking speed).

Together, this work seeks to yield a more accurate understanding of how neighborhood walkability is associated with network access and permeability, and to develop an easy-to-use interactive on-line tool for researchers and planners to modify neighborhood walkability to enhance access to features of interest. The aim of the research is to develop an open-source simple agent-based walkable network tool that complements existing walkability tools, by combining the benefits of open source road network service areas with scenario modeling. As such, this work will provide not only an innovative tool to investigate how neighborhood walkability is related to amenity access (e.g., TOD), but enable different planning scenarios to be simulated and tested prior to developing new or retrofitting older areas.

## Methods

### Study setting

This research formed part of the North West Metropolitan Region of Melbourne Data Access, Integration, and Interrogative and Demonstrator Projects [29] refer: http://www.csdila.unimelb.edu.au/projects/aurinands/aurinands_websitehtml/index.htm). Briefly, the aim of the project was to demonstrate the benefit of providing open access government datasets to researchers, planners and policy makers to deal with problems of space, place, and liveability in the North West Metropolitan region of Melbourne (Victoria, Australia). Four small-scale demonstrator projects were undertaken, focusing on key policy issues in the North West region of Melbourne; being access to affordable housing, health services, employment clustering, and walkability. Spatial datasets for the Melbourne North West corridor were made available through the Australian Urban Research Infrastructure Network (**AURIN**) portal and data repository (refer: http://www.aurin.org.au). The present paper focuses on the development of the basic agent-based walkable network tool created as part of the walkability demonstration project.

### AURIN

AURIN is a Super Science project funded by the Australian Government, tasked to provide e-infrastructure to support urban and built environment research, policy and decision-making within a national context. AURIN has developed an open source portal where users can come in and shop for spatial data, and analyze and visualize the results. Users can either run models within the portal or download the data and run them using other modeling tools. In this project the AURIN portal was used for accessing the necessary spatial data inputs for running the ‘what-if?’ scenarios in the North West Melbourne study area. The datasets accessed included the street network and features of interest; however additional data sources could be sourced externally and overlaid in future depending on the required tool complexity.

### Stakeholder working group and project champions

A stakeholder working group and industry- and research-based project champions were established as part of this research. The stakeholder working group comprised of representatives of Australian federal, state, and local government agencies drawn from transport, planning, and health sectors. This group met three times with the research team over the course of the project to: inform the content of the basic agent-based walkable network tool (Workshop I); provide interim feedback on the alpha version of the tool (Workshop II); and review the final assumptions, interface, and model capability (Workshop III). Industry- and research-based project champions further communicated the development and utility of the tool to a wider network, including government, academia, and industry.

## Results

### Model development

The initial development of the basic agent-based walkable network tool was informed by the health and place-based literature, and earlier related research undertaken by the investigators that applied and tested the walkability index with various health outcomes [9,30,31], along with a working ‘Ped-Catch’ prototype tool [32,33]. This earlier prototype was developed for pedestrian catchment analysis that utilized Autodesk’s Maxscript™ and PFlow™ within 3ds Max™ animation software. Although Ped-Catch has proved effective in design decision-making processes and is beneficial for design decision advocacy [34], it requires a high degree of skill to operate, proprietary animation software is used, and each site must be manually modeled specifically for the analysis.

Refinement of the Ped-Catch prototype was supplemented with information provided by the stakeholder-working group (Workshop I). Initial considerations included being able to utilize and upload a hierarchy of spatial data and the provision of editing and customization interface features to model different built environment and pedestrian scenarios. Bearing in mind the complexities in using the earlier tools, including the technical expertise required to manipulate these data; the basic agent-based walkable network tool was designed to be used with data drawn from the AURIN data repository as well as other open-access sources, and had relevant pre-specified user functionality built into the interface.

For the purposes of this paper, each agent represents a simulated pedestrian who travels along a network within a defined set of rules. We have created ‘simple’ agents to represent pedestrian movement throughout a network; however their behaviors were not based on survey data. The agents (i.e., simulated pedestrians) were programmed with simple rules to give them rudimentary pedestrian behaviors, including: moving at various speeds seeking to travel as far away as possible from a node within an allocated time; navigating a road or sidewalk network; being hindered by barriers, such as major roads or rivers; avoiding conflict with buildings and each other; and slowing down on steep topography. In other words, the simulated pedestrians leave from a user-defined node of interest, and traverse the street networks in all directions, unless blocked by an impassable network barrier. The catchment the simulated pedestrians can travel is further defined/constrained by the user setting a maximum time or distance (but not both parameters simultaneously). Together this results in an assessment of pedestrian destination accessibility for the defined conditions.

### Dataset availability

One objective of this tool was spatial data flexibility; that is, different users have the ability to upload data from diverse sources and at different scales. In order to do so, the tool was developed with a spatial data hierarchy in mind. Fine-grained data were optimal (e.g., the smallest census catchment area), but acceptable inputs extended to coarser-scale (e.g., suburb) and open-access (e.g., Walk score, Open trip planner) data sources. In this way, a variety of end users were able to utilize the tool either using their own data, or those supplied through the AURIN portal or other open-access sources. Standard spatial datasets used for the tool include the road network, features of interest (e.g., schools, public transport nodes), and traffic lights. Depending on end user access to other datasets, the tool was designed to support additional spatial layers. These could include (but were not limited to) sidewalks, traffic volume, and topography. Including such additional spatial data layers enhanced the accuracy and relevance of the tool for policy-makers and practitioners, as well as the ability to adapt to different environments.

### User-specified functionality

The tool was developed to assess and optimize the walkability of neighborhood catchments around actual or potential nodes of interest (e.g., schools, public transport stops) based on access according to street network connectivity and the population of interest (e.g., vulnerable populations, such as children or older adults). In order to achieve this, a series of user-specified functionalities were designed into the interface. These included sliding bars to manipulate the: maximum walking time (up to 20 minutes), maximum walking speed (up to 2 m.s^-1^), and intersection wait time (up to 60 seconds). All of these attributes are theoretically linked to walking behaviors [35]. For example, walking and road crossing speeds vary greatly by different ages and levels of mobility [36,37], and thus, having the ability to alter these was an important feature of the tool. It was hypothesized that destinations for more vulnerable groups (e.g., children and older adults) would have smaller walkable catchments and they would access fewer crossings because of relatively slower walking speeds and additional safety concerns, respectively, when compared with able-bodied adults. This type of information is critical for assessing the walkable catchment area for destinations accessed by different population groups (e.g., schools, senior citizen organizations). While such user-specified functionalities are not new to agent-based modeling [27], these attributes have been identified as important considerations for creating walking-supportive environments [35], and in turn, increases the relevance of the findings for planning and public health disciplines.

**Figure 1:**
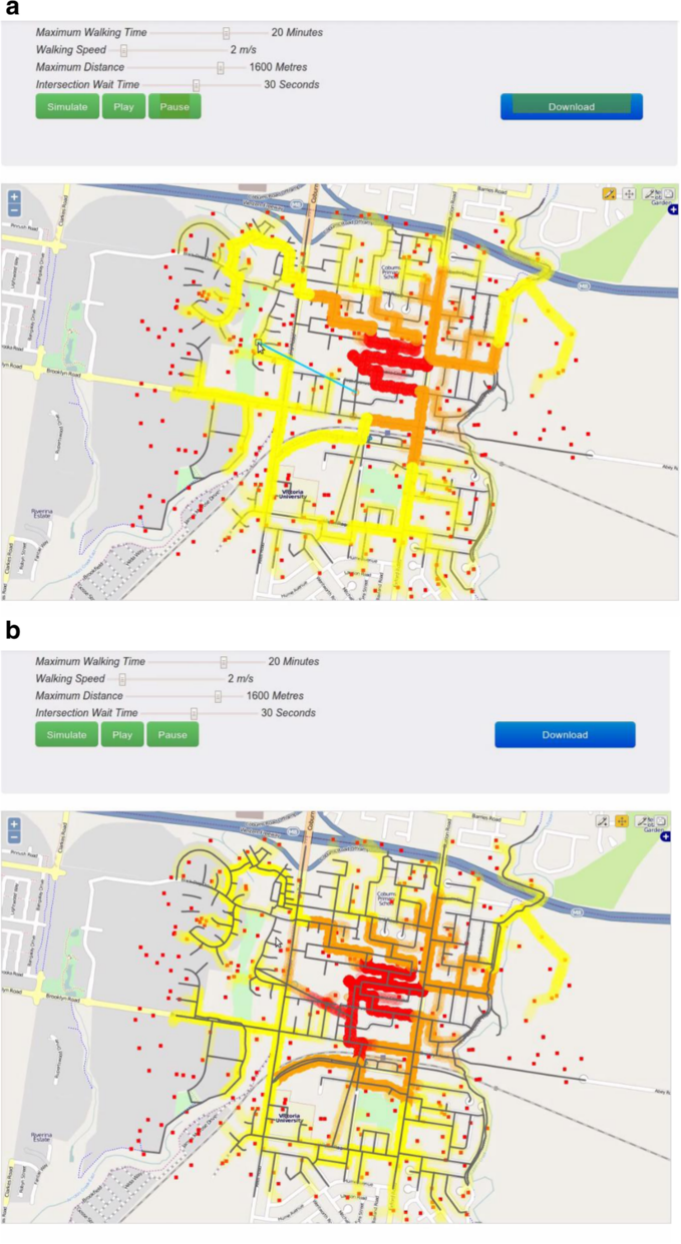
**Screenshots showing the vector editing functionality of the simple agent-based modelling tool. a** shows the vector addition in blue. **b** shows the agents traversing the new network.

Vector editing functionality allowed users to assess and optimize walkability catchments around origins by adding or removing networks to modify street connectivity, and manipulate the simple agents’ starting point to reflect potential origins of interest (e.g., public transport egress, a school, a retirement home). This allowed different scenarios to be modeled within a simulated environment. An example of this is shown in Figure 1, where the user has added a blue line in Figure 1a; Figure 1b shows the agents travelling the new connection.

### Simulating and visualizing the data

**Figure 2:**
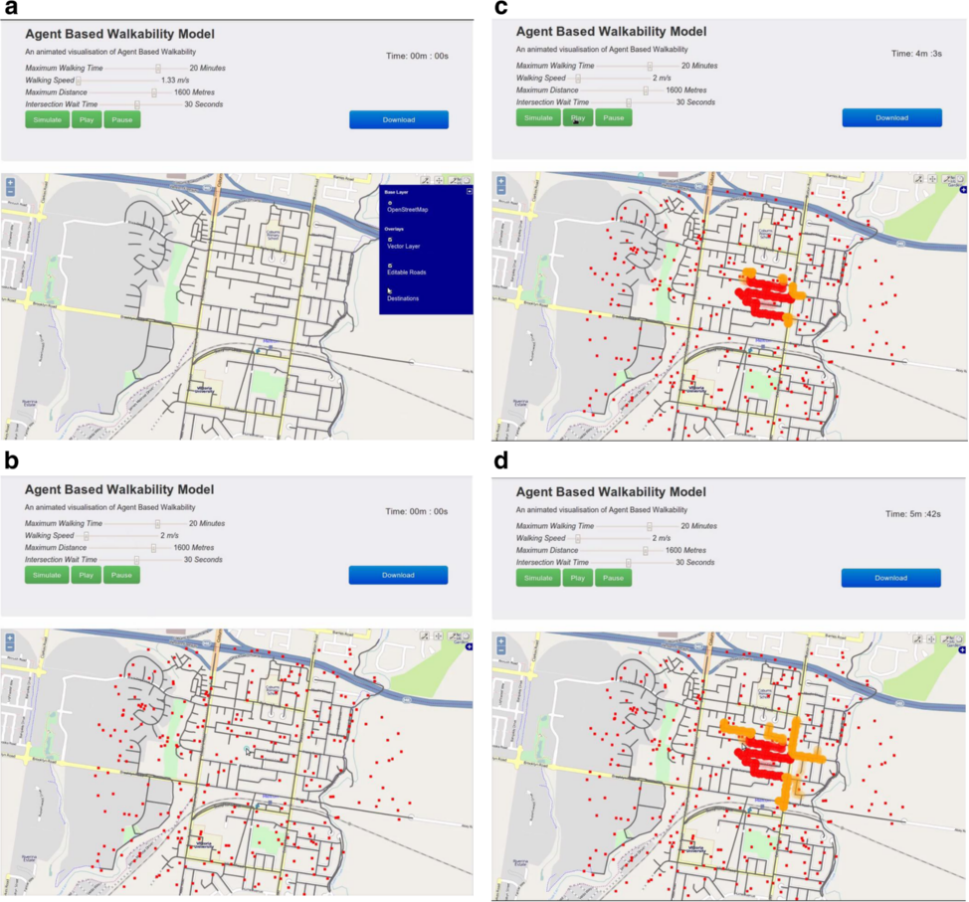
**Screenshots of the simple agent-based modelling tool interface and agent movement over time. a** shows the interface functionality. **b** shows the distribution of destinations across the network. **c** and **d** show agents dispersing through the network with the graded colours indicating agent density.

The simulation components were written in Java using the open source GeoTools Graph library (OSGeo Foundation, Chicago, IL), and for visualization a web-based client was built around the open source OpenLayers (OSGeo Foundation, Chicago, IL) mapping tool. A network dataset (representing roads or sidewalks) using GeoTools functionality was ingested in a geo-referenced vector file format to enable simple agents to perform graph traversal operations using the A-Star navigation algorithm [38]. In addition, using JTS (VividSolutions Inc, Victoria, BC) (the core geometry library used by Geotools), the network dataset for the simulation was prepared by noding intersections and snapping the origin and destination points to the road network. This allowed simple agents to traverse from origins to destinations that were proximal, but not directly on the road network. The paths the agents followed from origin to destination were calculated using this enhanced network and the A-Star algorithm [38]. These paths were converted into a series of equally spaced points with a timestamp attribute, taking into account time spent waiting at intersections. The final step was to extract this information into a geo-referenced file format (GeoJSON (OSGeo Foundation, Chicago, IL) or ESRI (ESRI, Redlands, CA) shape files). Once a time-stamped point dataset was generated, visualizing in OpenLayers was a relatively simple process using OpenLayers time filter animation. This method filtered the dataset to show slices of time incrementally, as demonstrated by the screen shots below (Figure 2a-d).

### Broader model considerations

The simple agent-based walkable network tool was designed in an interdisciplinary research environment (i.e., public health, urban planning, geomatics, geography) for a multi-disciplinary and multi-sectorial audience (e.g., urban designers, planners, policy and decision-makers). As such, it also needed to function in hardware within a standard computer figuration (i.e., low computational power); and provide an interface that was easy for non-spatial specialists to navigate. Thus, the tool was initially developed using simple agents with a limited level of artificial intelligence; that is, agents had a set of rules and behaviors initially programed. Conversely, complex agents are those who continue to learn behaviors from subsequent interactions with the environment and other agents [23]. In our model agents left from a user-specified node of interest (origin) and navigated according to simple behavioral rules toward randomly distributed points snapped to the road network within the parameters set by the user (e.g., walking speed, time, intersection wait time) (see Figure 2b). Having limited artificial intelligence ensured different ‘what-if’ scenarios could be rapidly tested (e.g., different road network configurations or origin of agents). The stakeholder group regarded this functionality as being important.

### Accessing the tool

The final basic agent-based walkable network tool is currently available at: http://155.146.87.16:999/agent-walkability/agent-model.html, and a movie tutorial can be found at: http://blogs.unimelb.edu.au/aurinands/2013/05/29/walkability-demonstration-of-value/. Source code is available from the GitHub public repository: https://github.com/gusmacaulay/agent-walkability. The intention is to integrate the final tool into the AURIN portal.

### Metrics

Based on potential future research applications and stakeholder feedback, a series of metrics were generated after each model simulation was run. These outputs include: a visual, graded representation of agents throughout the network; area coverage comparison between the agent-based model and circular catchment expressed as a ratio (i.e., pedshed); and the mean number of intersections the agents crossed. All of these outputs are recognized as being important attributes of walkability [35], which in turn are associated with walking behaviors and health outcomes [39,40]. Metrics were generated as .csv and shape files after running each model, thereby allowing comparisons of walkability to be made across different built environments locations and populations of interest.

## Discussion

This simple agent-based walkable network tool leverages off the substantial body of earlier walkability and agent-based modeling work. To our knowledge, this is the first open-source GIS tool that allows street network interventions to be tested and evaluated, and the walkable catchment area to be assessed via an animated agent-based simulation within a web interface. This is important as built environment interventions are costly to implement; however, the longevity of such modifications coupled with the large number of people exposed to urban settings make these cost-effective public health interventions in the long-term if done correctly from the outset [41]. The multidisciplinary and multi-sectorial group of stakeholders recognized the need for a tool that provided the opportunity to trial and evaluate different scenarios focused on walkability metrics prior to building infrastructure. Features of the tool that were highly regarded by this group were the rapid testing of multiple scenarios, data flexibility, inbuilt user specifications, and metric outputs. Indeed, end users have already applied the tool to model potential train station nodes and potential pedestrian walkways in the North West Metropolitan region of Melbourne.

The ability to design environments that facilitate walking from home to local employment and education, shops, services, and public transport has important far-reaching implications for social determinants of health. Globally, physical inactivity accounts for approximately 9% of deaths per year (~5.3 million people) attributed to non-communicable diseases [42]. Therefore, modest increases in walking across populations will generate substantial health benefits [43], as well as broader upstream gains if the environment supports access to important infrastructure such as local employment, health care, and education [44]. Furthermore, higher levels of walking have been associated with increased social cohesion (an important predictor of mental health) [45], and reduced reliance on motor vehicles, thereby reducing greenhouse gas emissions [46] and minimizing fuel vulnerabilities [47,48]. These issues are of critical importance as ‘affordable’ housing green-field developments are frequently located on urban fringes. Such developments often have limited access to public transport, shops and services [49] resulting in higher levels of car dependency, increased vehicle miles travelled, and lower levels of active forms of travels for residents of urban sprawl [50]. Furthermore, although confirmatory longitudinal studies are still needed for certainty, emerging evidence suggests a causal relationship with the built environment influencing physical activity behaviors [51-53]. Therefore supposing the model assumptions are valid, we can reasonably expect that creating more walkable environments, either virtually or physically, will lead to increases in simulated or actual walking levels, respectively.

Although agent-based modeling is not a new concept, its use is not widespread in many disciplines, including public health; therefore there may be some initial conceptual challenges when people from other disciplines are introduced to the tool. However, because of its predefined interface functionality and link to open source datasets, the tool is fairly simple to manipulate for those without geographical expertise. Nevertheless, many challenges existed for this simple agent-based walkable network tool, including being able to: 1) source and utilize a wide range of spatial data, where access may differ between users; 2) be operationalized by users with limited spatial or software engineering expertise; and 3) ensure meaningful outputs that relate to multidisciplinary policies and practices. As such, the tool was designed to accommodate a range of data and was developed through an interdisciplinary collaboration to ensure that outputs were useable and informative.

The uptake of the tool is dependent upon spatial data availability and the sustainability of the AURIN program; however there is potential for users to upload their own open-source data layer. This reduces the risk of road network data being unavailable. However, we faced substantial challenges when attempting to access more refined data layers (e.g., sidewalks). For example, sidewalk data were available in some, but not all of the trial areas. When sidewalk data were available, they did not connect to centerlines as they do with a road network; therefore sidewalks would need to be manually connected to centerlines before uploading into the tool. As such, additional resources will need to be allocated as the complexity of the tool increases. Having the ability to import more detailed sidewalk data into the tool will support the development of a pedestrian network, providing greater accuracy in modeling pedestrian movements. This has prompted the Municipal Association of Victoria to develop a working group to standardize and optimize sidewalk data collection across the region.

### Next stages

There is much scope to increase the sophistication and relevance of this tool. First, currently the tool only includes a limited number of objective measures of the environment. Other measures could be explored, such as crime, incivilities, aesthetics, greenery, and traffic noise, both objectively and subjectively. Each of these variables have been associated with the likelihood of walking [35], and research has shown substantial differences can exist between objective and subjective (perceived) measures of crime and incivilities [54]. In future such measures could be modeled, with changes to agents’ walking behaviors informed by research based on level of exposure for a given environmental attribute. For example, separate models could be tested to explore different associations for distinctive populations (e.g., women’s walking levels decline at a lower perceptions of crime threshold than men’s [54]). Second, as mentioned previously, importing clean sidewalk data would enable the pedestrian network to be modeled, rather than using the road network, and this would provide a more accurate representation of pedestrian flow. Third, this tool is presently deployed as a simple model, but future versions could extend to complex agents. This could include training agents’ movements by calibrating and validating behaviors based on survey data and creating a synthetic population for a given study area. Complex agent-based models have been used to simulate land use and transport dynamics in urban environments (e.g., UrbanSim, TransSim, MATSim) [55-57], but thus far have not considered walkability. As such, our research provides an important first step in building an agent-based model ‘walkability’ simulation component that could in future form a module in one of these more comprehensive toolkits. Fourth, it remains to be tested whether simple agents’ behaviors across the different virtual interventions translate to similar pedestrian behaviors in a real-world setting. By using tools such as global positioning systems (GPS), it is now possible to compare free-living pedestrian behaviors with simple agents’ navigation of virtual environments prior to staging an intervention. These GPS data could then be used to test the validity of model assumptions and refine the agent-based model assumptions, especially in relation to urban characteristics known to influence walkability (i.e., residential density, land use mix, street connectivity) [6-8]. Last, this tool has been designed to be used in conjunction with an open-source ‘walkability index’ [58] as part of a ‘walkability toolkit’ that will be housed in the AURIN portal. Therefore, these tools have been designed to: first, identify the existing walkability of a given area (walkability index); and second, to examine how these areas can be manipulated to modify the walkable catchment area (basic agent-based walkable network tool).

## Conclusions

To conclude, this simple agent-based walkable network tool has the potential to be not only a powerful urban design tool that builds on existing walkability measures, but also an influential planning and public health advocacy tool. The open-access nature of the tool means that it is available to those who have access to a standard computer configuration and modern internet browser. This project demonstrates the benefits of bringing together multiple data sources, disciplines, and sectors to respond to challenges faced in urban planning and policy. Furthermore, there is much scope to extend this tool, including incorporating diverse spatial and non-spatial data and integration with other tools developed through this project and the more commonly used walkability index. Together, this has much potential to facilitate the development of more walkable and accessible neighborhoods, and around key destinations of interest.

## Abbreviations

ANDS: Australian national data service; AURIN: Australian urban research infrastructure network; GIS: Geographical information systems.

## Competing interests

The authors’ declare that they have no competing interests.

## Authors’ contributions

HB drafted the manuscript; SE, HB and MW managed the project; GM developed the tool; and all authors provided intellectual contributions for the tool development, critically revised manuscript drafts, and read and approved the final version of the manuscript.
